# Inferring subjective states through the observation of actions

**DOI:** 10.1098/rspb.2012.1847

**Published:** 2012-10-03

**Authors:** D. Patel, S. M. Fleming, J. M. Kilner

**Affiliations:** 1 Sobell Department of Motor Neuroscience and Movement Disorders, IoN, UCL, London WC1N 3BG, UK; 2 Center for Neural Science, New York University, 4 Washington Place, NY 10003, USA; 3 Department of Experimental Psychology, University of Oxford, South Parks Road, Oxford OX1 3UD, UK

**Keywords:** inference, kinematics, action observation

## Abstract

Estimating another person's subjective confidence is crucial for social interaction, but how this inference is achieved is unknown. Previous research has demonstrated that the speed at which people make decisions is correlated with their confidence in their decision. Here, we show that (i) subjects are able to infer the subjective confidence of another person simply through the observation of their actions and (ii) this inference is dependent upon the performance of each subject when executing the action. Crucially, the latter result supports a model in which motor simulation of an observed action mediates the successful understanding of other minds. We conclude that kinematic understanding allows access to the higher-order cognitive processes of others, and that this access plays a central role in social interactions.

## 1. Introduction

Sharing of subjective confidence is suggested to be critical for group decision-making [1,2]. However, how individuals infer each other's confidence in decision-making has not been addressed. Previous research has shown that the speed at which the subject makes a forced choice decision is correlated with their confidence, with reaction times being faster for more confident decisions ([3], electronic supplementary material, figure S3). One prevalent notion is that reaction times provide an internal cue as to the difficulty of the decision, such that longer reaction times tend to indicate lower confidence decisions [4]. In an evidence accumulation framework, reaction times naturally vary with the strength of evidence supporting one or other choice and noise in the accumulation process, both of which are predictors of the correctness of the decision [5,6]. We hypothesized that reaction time might serve as a useful cue for the inference of confidence in the decision-making of others. Here, we ask whether simply observing another individual's actions, in lieu of explicit communication, is sufficient for inferring subjective confidence.

Interest in action observation has grown in the last two decades, in part owing to the neurophysiological discovery of mirror neurons in the monkey ventral premotor cortex and inferior parietal cortex. These neurons discharge  when the monkey performs specific hand movements and also when it observes a human performing the same movements [7–10]. Many believe that mirror neurons provide a conduit to ‘turn visual information into knowledge’ [11,12]. Indeed, it has been proposed that one's actions are intrinsically linked to perception, and that imagining, observing, preparing, or in any way representing an action excites the motor programmes used to execute that same action [13,14]. Action observation can be described at many different levels: the overall intention of the action, the short-term goals required to realize the overall intention, and the kinematics of the action, or how the hand and arm move through space [15,16]. The majority of previous research into action observation has focused on the role of the motor system in inferring the goal or intention of the observed action [12]. More recently, it has been shown that subjects are also sensitive to subtle changes in the kinematics of an observed action [17–23]. In the current study, we ask whether subjects correctly infer another person's confidence simply through the observation of their actions. To the extent that this inference is mediated by the action observation system, we hypothesize that confidence will be judged as relative to the observer's own actions. Our results reveal a mechanism by which important social information required for optimal group decisions could be shared between individuals without explicit communication.

## 2. Methods

### (a) Participants

Seventeen subjects were recruited for the study, 10 males and seven females, with a mean age of 21 (range, 20–26). Subjects were recruited from the University of London. All subjects gave signed consent and the study was approved by a local ethical committee. Of the 17 subjects, two subjects, one male and one female, performed only one of the tasks—the execution task. The actions of these two subjects were video recorded with their consent and data from these videos were used in the second task—the observation task. The remaining 15 subjects performed first an execution task and then an observation task.

### (b) The execution task

The experiment involved a contrast discrimination task identical to that used by Fleming *et al.* [3]. Subjects were shown two images in swift succession on a computer screen. Each image consisted of six Gabor gratings (circular patches of smoothly varying light and dark bars) arranged around a central fixation point. The background was a uniform grey screen of luminance 3.66 cd m^−2^ (see electronic supplementary material, figure S1*a*). In one of the two images, all of the Gabor gratings were set at the same contrast (‘baseline Gabors’), but in the other image, one of the Gabors was set to be a higher contrast than the other five ‘baseline’ Gabors, and appeared as a ‘pop-out’. ‘Baseline’ Gabors were displayed at a contrast of 20 per cent (where 0% was not a visible difference between the light and dark grating bars and 100% is the maximum difference). The ‘pop-out’ Gabors varied in contrast between 23 and 80 per cent, in increments of 3 per cent. The appearance of the ‘pop-out’ Gabor in either the first or second image, its contrast and its spatial position (orientation around the central point) in each trial varied randomly throughout the experiment [3].

After presentation of the two images, the subject was required to make a decision as to which image (first or second) they believed contained the ‘pop-out’. After each decision, subjects were asked to rate their confidence on a scale of 1–6 (1 denoting lowest possible confidence). The participants were required to express their choice by using their dominant hand on a custom-made response board (see the electronic supplementary material, figure S1*b*). The board comprised four separate sensors: a sensor on which the hand rested between each trial, a sensor on which a marble was placed, and two sensors, each within holes equidistant from the resting position of the marble that sensed when the marble was placed into the hole. After the presentation of the two images a grey screen appeared with the numbers ‘1’ and ‘2’. To convey their forced decision as to which image contained the ‘pop out’ Gabor, the participant was required to move the marble from its rest position in the centre of the board and place it on either the left or right hole, corresponding to the first or second image, respectively. Subjects were given no instruction as to how fast or slow to move the marble. Depending on where the marble was placed (after the marble and hand are returned to their original rest positions on the board), a red square frame appeared around either ‘1’ or ‘2’ on the computer screen to highlight the participant's decision. Following this, an additional grey screen with the numbers ‘1’–‘6’ appeared, requiring the participant to rate their confidence in the decision they have just made on a scale of 1–6 (1 being least confident). This rating was expressed using the numerical keys of the QWERTY keypad on the laptop using their non-dominant hand (i.e. the hand that is not placed on the sensor), and subsequently a red square frame appeared around the selected rating.

The contrast of the ‘pop-out’ Gabor was adjusted throughout the experiment using a two-up, one-down staircase procedure such that all participants converged onto a final score of approximately 71 per cent correct (correct referring to making the right decision as to which of the two images contained the pop-out). The staircase operated such that after two consecutive correct decisions the contrast was decreased by one step, whereas after one incorrect decision the contrast was increased by one step. This was done to ensure that the analysis of movement time (MT) and confidence was not affected by performance. It also helped one to ensure that subjects used the full extent of the confidence scale. Fifteen subjects performed 50 trials of this task in one block. The two subjects whose actions were recorded performed four blocks of 50 trials making 200 trials in total for each of these subjects.

### (c) The observation task

In this task, subjects were asked to watch a series of video clips showing the hand movements of the two anonymized actors (one male and one female) during the execution task. All video clips were edited to start 300 ms prior to onset of the movement. After watching each clip, subjects were asked to rate how confident they felt the individuals in the videos were in their decisions in the same way as in the execution task. No feedback was given for this task. Ten of a possible 400 trials were omitted, as they were longer than 6 s in duration. The experiment therefore consisted of 390 trials that were presented in a random order. Subjects performed three blocks of 100 trials and one block of 90 trials, with an interval between each.

### (d) Data analysis

Data for two subjects were not analysed as they did not understand the task instructions for the execution task leaving 13 subjects for further analysis. The first 10 trials were excluded from analysis to allow for adaptation to the task. All trials in which the movement was >6 s were removed from further analysis. For the execution data, we correlated four different time intervals of the movement with the subjects' rating of confidence across trials. These intervals were the response time (RT), the time the subject began to move, the pick up time (PT), the time when the subject picked up the marble and the end time (ET), the time when the marble was placed in either the left or right response hole. In addition, we calculated the MT, the difference between the ET and the RT. All timing measures were log-transformed prior to analysis to render them normally distributed. For each subject, we calculated the gradient of the linear dependency between the four timing measures and confidence. In addition, we calculated the linear dependency between the mean MT and the mean confidence level across subjects. The gradient of these linear correlations was then used as summary statistics for each subject.

For the observation task, we calculated the linear dependency between MT and the observer's confidence (oCon) and between the actor's confidence and the oCon. As before, we used the gradients of the linear correlations as our summary statistics for each subject. To assess whether there was a dependency of the parameters of execution on perception we performed three analyses. First, we asked whether there was a relationship between the difference in mean inferred confidence (iCon) and mean performed confidence (pCon) and the difference in the mean observed MT (oMT) and the mean performed MT (pMT) (see electronic supplementary material, figure S2). This measure was used to test the hypothesis that, on average, subjects inferred the confidence of the actor relative to how they performed the action. Second, we asked whether the linear dependency between MT and confidence was the same for the execution and observation tasks. To this end, we performed a correlation between the linear gradients of each subject for the execution and observation conditions. If subjects used their motor system to infer the confidence from the observed action, then one would predict that the relationship between pCon and pMT will be the same as that between the iCon and the oMT (see electronic supplementary material, figure S2). Finally, we made a prediction of the mean iCon from the oMTs and correlated this prediction with the actual mean iCon. The predicted mean iCon was generated using only the parameters of each subject's MT–confidence regression equation.

### (e) Analysis of metacognitive sensitivity to others' decisions

Here, we used a non-parametric estimate of metacognitive sensitivity that characterized the probability of being correct for a given level of confidence. Receiver-operating characteristic (ROC) curves were anchored at [0, 0] and [1, 1]. An ROC curve that bows sharply upwards indicates that the probability of being correct rises rapidly with confidence; conversely, a flat ROC function indicates a weak link between confidence and accuracy. To plot the ROC, hi = p(confidence = i |correct) and fi = p(confidence = i | incorrect) were calculated for all i. These probabilities were then transformed into cumulative probabilities. The area underlying the ROC curve (*A*_ROC_) was calculated by the sum of the area between the ROC curve and the major diagonal and the area of the half-square triangle below the major diagonal:2.2
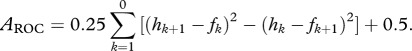
Data shown here are available at doi:10.5061/dryad.q0k1m

## 3. Results

### (a) Relationship between confidence and kinematics when executing the action

**Figure 1.**
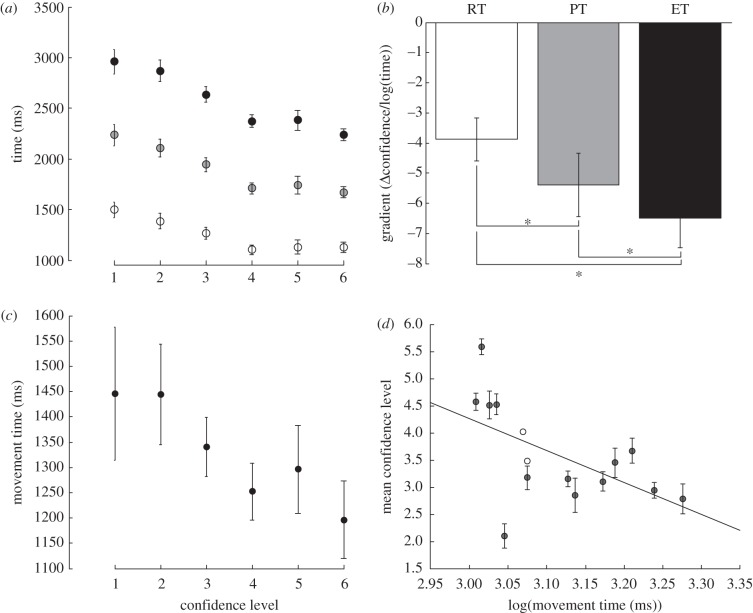
Action execution. (*a*) The average time for three of the timing measures, RT (open circles), PT (grey circles) and ET (black circles) across subjects for the six confidence levels. (*b*) The mean gradient from the linear correlation of confidence level with the log-transformed timing data for RT (open circles), PT (grey circles) and ET (black circles) across subjects. Asterisk indicates significant differences at *p* < 0.05. (*c*) The mean movement time, MT, averaged across subjects for each confidence level. (*d*) The relationship between mean MT and mean confidence for each subject. In each panel, errorbars show s.e. of the mean.

In keeping with previous studies [3], we showed that the subjects' level of confidence in their decision was correlated with their RT (figure 1*a*, open circles). Across subjects, the slope of the regression between confidence and the log of the RT was significantly negative (*t*_12_ = −5.43; *p* < 0.05); in addition, nine of the 13 subjects showed a significant correlation (*p* < 0.05) between confidence and log RT. Crucially, there was also a significant correlation between both PT and ET and the subjects' level of confidence (*t*_12_ = −5.12; *p* < 0.05, *t*_12_ = −6.54; *p* < 0.05 for PT and ET**,** respectively: figure 1*a,b* grey and black circles and bars). PT and ET had a greater effect on subjects' confidence than the RT measure, with the average slope being significantly more negative for both PT and ET than RT (*t*_12_ = −3.86; *p* < 0.05; and *t*_12_ = −7.43; *p* < 0.05 respectively). Further, the effect of ET on confidence was significantly greater than PT (*t*_12_ = −3.84; *p* < 0.05). This result demonstrates that subjects' confidence is not only reflected in the time it takes to respond, but also is reflected in the speed of the movement once it has been initiated. Such kinematics may provide a social cue to confidence.

As the aim of this study was to relate parameters of the kinematics all further analyses were restricted to the MT, defined as the difference in time between the ET and RT measures. Therefore, for performed actions the key parameters were the pMT  and pCon. There was a significant effect of subjects' pCon on pMT (figure 1*c t*_12_ = −4.75; *p* < 0.05). Having shown that there was a significant relationship between subjects' confidence in their decision and their MT, we investigated whether a similar relationship was present across subjects. In other words, we hypothesized that subjects who are on average less confident in their decision tend to move slower than those who are more confident. This is what was found. Across subjects, there was a significant correlation between subjects mean pCon and their mean pMT (figure 1*d*; *R*^2^ = 0.32, *p* < 0.05).

### (b) Relationship between confidence and kinematics when observing an action

**Figure 2.**
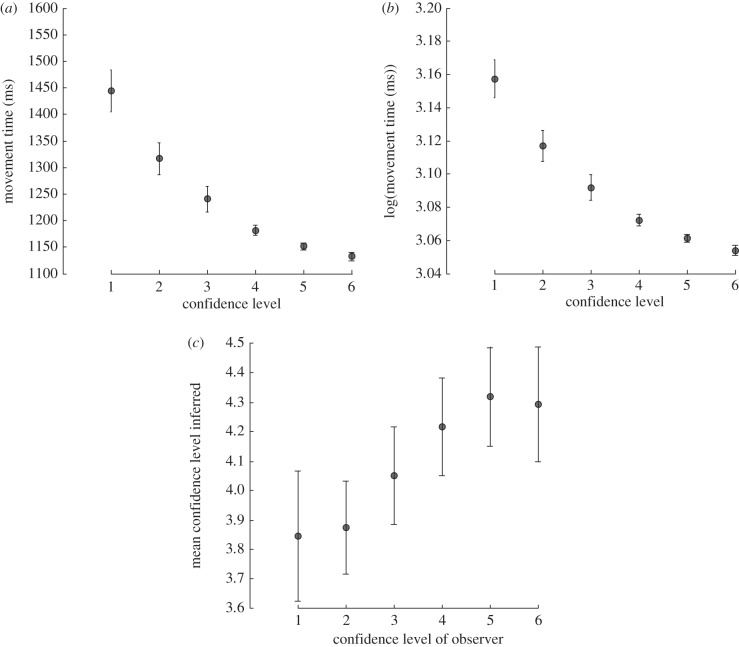
Action observation. The plots of the mean MTs of the observed actions that were rated at the six different confidence levels. (*a*) The raw average MT and (*b*) the same data for the log-transformed MTs. (*c*) The relationship between the average confidence ratings of the person executing the action and those inferred by the observer. In each panel, error bars show s.e. of the mean.

The results of figure 1 establish a significant and consistent relationship between kinematic parameters of a reach and grasp action and subjects' confidence in their decision. We next asked whether observers harnessed this relationship to infer another actor's level of decision confidence when observing their actions. To this end, we asked subjects to observe a series of video clips showing one of two people performing the same discrimination task described above. The video clips showed only the reach, pick up and decision part of the task. All video clips were edited so that they had the same RT of 300 ms. After each video observers were asked to judge the actor's decision confidence in the same way as before (iCon). Therefore, for observed action the key parameters were the oMT, the oCon and the iCon. Across subjects, there was a significant correlation between the oMT and the iCon rating (figure 2*a*,*b*; *t*_12_ = −9.57, *p* < 0.05). In addition, 12 out of 13 observers demonstrated a significant correlation (*p* < 0.05) between the oMT and their iCon level. Importantly, there was also a significant correlation between the actor's oCon rating in their decision and that estimated by the observer, iCon (figure 2*c*; *t*_12_ = 9.21; *p* < 0.05).

### (c) Relationship between perception and action

**Figure 3.**
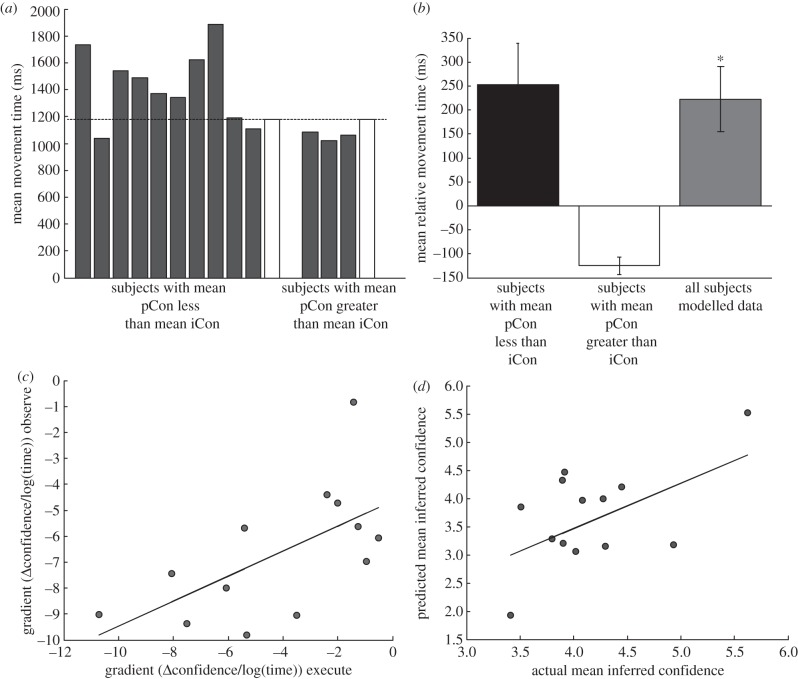
Relationship between action execution and perception. (*a*) The mean MTs for each subject. The grey bars show the data for those subjects who performed the observation task whereas the white bars show the mean MT for the two subjects whose actions were recorded for the observation conditions. The data on the left shows the data for subjects whose mean confidence rating was less than the mean confidence ratings of the observed actions and the data on the right shows the data for subjects whose mean confidence rating was more than the mean confidence ratings of the observed actions. (*b*) The mean difference in MTs across subjects for subjects with mean confidence less than that of the observed actions (black bar), subjects with mean confidence more than that of the observed actions (white bar) and the modelled data across all subjects where the data for the white bars is multiplied by −1 (grey bar). Asterisk indicates a significant effect *p* < 0.05. (*c*) The plot of the gradient of the relationship between MT and confidence for the execution and observation conditions for each subject. (*d*) The relationship between the actual mean inferred confidence and *predicted* mean inferred confidence for each subject.

The previous results demonstrate that when observing someone else's action, it is possible to infer their level of confidence in the decisions made. However, the fact that there is a correlation between these metrics does not mean that the observer used their motor system to infer the level confidence. One alternative strategy would be for the observer simply to rate faster trials as more confident and vice versa. Indeed, the majority of subjects reported in a post-experimental debrief that they noted that some movements were faster than others. To test whether there was a statistical link between action and perception, we asked whether subjects judge actions relative to how they would have performed the same action. In other words, are subjects who rate observed decisions as having greater confidence than their own decisions also slower than the average MT on the videotape? This was indeed the case. Of the 10 subjects who rated the actor as more confident on average than their own decisions, eight of them moved more slowly than the actor. Of the three subjects who rated the actor as less confident than themselves, all three moved faster than the observed actions (figure 3*a*). Therefore, 11 out of the 13 subjects had behaviour consistent with judgements of confidence being relative to one's own actions (*p* < 0.05 Sign-test; *t*_12_ = 3.27, *p* < 0.05; figure 3*b*). This provides evidence that a subject's model of how to execute the action is employed when observing others' actions. To further test this claim, we investigated the relationship between the impact of MT on confidence during execution and observation. We hypothesized that if subjects used a model of their own actions when inferring the confidence of others, then MT–confidence slopes should positively be correlated across action and observation conditions. This is precisely what we found. The slopes describing the relationship between MT and confidence were significantly correlated across conditions (*R*^2^ = 0.37, *p* < 0.05; figure 3*c*). Finally, we generated a prediction of each subject's iCon level solely from their confidence–MT relationship during the execution task *given the* oMT (from subject-specific linear regression parameters). We found that a simple prediction derived from movement execution parameters accounted for 25 per cent of the variance in iCon ratings, reaching trend-level significance (*R*^2^ = 0.25, *p* = 0.07; figure 3*d*).

### (d) Relationship between perceived confidence and observed decision performance

**Figure 4.**
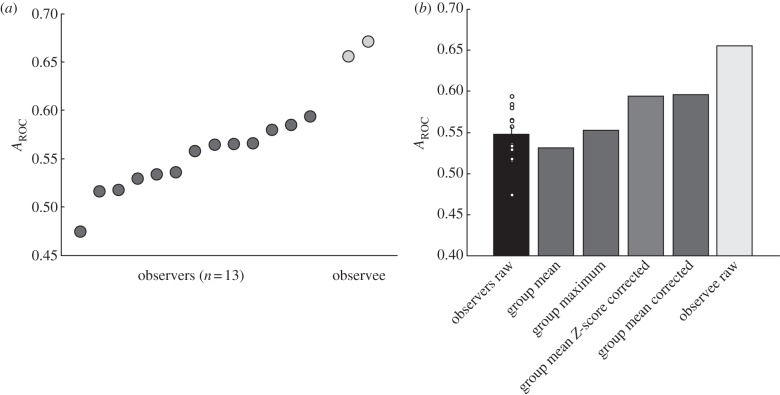
Relationship between inferred confidence and metacognition. (*a*) The *A*_ROC_ measure for each of the 13 subjects when observing the decisions of an actor (dark grey circles). The light grey circles show the same *A*_ROC_ measure for the two actors. (*b*) The mean of the *A*_ROC_ measure across subjects (individual measures are shown as open circles). The grey bars show a group *A*_ROC_ measure calculated using four different methods (see the text). Finally, the mean *A*_ROC_ for the actors is shown.

All the previous analyses focussed on the observers' ability to infer the confidence of the actor. We next asked whether the iCon is sensitive to whether the actor's decision was correct or incorrect. We adapted a measure of this sensitivity, *A*_ROC_, previously employed to investigate subjects' ability to monitor their own decisions [3]. Across observers, this *A*_ROC_ measure was significantly greater than chance (*A*_ROC_ = 0.5; *t*_12_ = 5.63, *p* < 0.05; figure 4*a*,*b*). Although significant, the metacognitive sensitivity of the observer was less than the sensitivity of the actor (*t*_12_ = −13.6; *p* < 0.05; figure 4*a*,*b*).

We additionally asked whether pooling iCon across the group could improve on the metacognitive sensitivity of any given individual. We tested four models of group decision-making by calculating for each trial a ‘group’ confidence rating that was either (i) the mean confidence level across subjects; (ii) the maximum confidence value across subjects; (iii) the mean confidence level having first mean-corrected each subject's confidence level across trials; and (iv) the mean confidence level having first *z*-scored each subject's confidence level across trials. Of these measures, the two corrected confidence levels showed a significant improvement on individual *A*_ROC_ measures. Indeed, the group mean-corrected *A*_ROC_ measure outperformed any of the 13 individual *A*_ROC_ measures (figure 4*b*).

## 4. Discussion

Here, we demonstrate that people are able to correctly infer the subjective confidence of another person simply from the kinematics of their observed action. In addition, we show that the relationship between the kinematics of the observed action and inferred subjective confidence could be explained by each individual subject's confidence–movement speed relationship. These results are consistent with the idea that the subjects employed their own motor system to make an inference on the observed actions.

In the last two decades, there has been a large body of research investigating the role of the motor system in action understanding [11,12,15,16,24–27]. However, there is no real consensus on whether ‘understanding’ during action observation depends upon activity in the motor system [11,12,15,16,24–27]. Ever since the discovery of mirror neurons in area F5 of the macaque monkey, the majority of research has focussed on the role of the motor system in inferring the goal or the intention of the observed action. However, there is little compelling evidence in support of this functional role [24,25]. In particular, there is very little evidence that has shown a correlation between people's ability to correctly perceive the goal and their ability to execute an action with the same goal. Here, we have shown that variance in subjects' perception of others' subjective states can be explained by variance in the way subjects execute their own actions. Rather than making an inference based on the goal or intention of the observed action, here subjects made their inference based on the kinematics of observed action.

The kinematics of an action is a relatively low-level description of the action, and it is not immediately obvious why it would be of functional importance to ‘understand’ the observed action at this level. However, we know that parameters of the kinematics of an action, for example RT, are modulated by a wide range of higher-order cognitive processes as evidenced by the fact that mental chronometry is one of the standard tools of cognitive psychology [28]. One possibility is that by ‘understanding’ the action at the kinematic level, we have access to these higher-order cognitive processes—here, the confidence of the actor that their decision was correct. The importance of action understanding and inference at the level of the kinematics is supported by studies that have demonstrated that subjects are able to make high-level inferences based only on changes in the kinematics of the observed action [23,29,30].

A predictive coding model of action understanding holds that the motor system and mirror neurons generate a prediction of the kinematics of the observed action, rather than being driven by the observed action itself [16,17,31]. To date the majority of work providing evidence for these models has focussed on the inference of the goal of the action [32,33]. Here, we demonstrate a systematic relationship between subjective confidence and action kinematics. Furthermore, and consistent with a predictive coding account, our results reveal that the observer assesses the confidence of an observed action relative to how they themselves would execute the same action. Firstly, we have shown that observed actions that were slower than subject's own actions were ascribed less confidence (figure 3*a*,*b*), Secondly, we have shown that the relationships between MT and confidence under observation and execution conditions were correlated across subjects (figure 3*c*,*d*). Within this framework, one possibility is that an observer makes a prediction of the kinematics of the action as if they were going to perform the action using their own motor system. This prediction is then compared with the actual kinematics. If the action is faster than predicted, observers rate the action as more confident, and if it is slower, then they rate the action as less confident. The degree to which confidence is modulated by the difference between the predicted and actual kinematics depends upon the observer's internal relationship between confidence and MT.

Although the results are consistent with the role of the motor system in inferring confidence from an observed action, there are other possible explanations of the results. One possibility is that the perceptual inference is achieved by purely visual discrimination. Indeed, this is possible if subjects have learnt the mapping between the kinematics of the observed actions and confidence. However, it should be noted that the kinematics of an action are dependent not only on confidence but also on many other factors including object properties such as the shape, size and texture. Therefore, any purely visual mapping between the kinematics and the confidence would also have to accommodate differences in the kinematics that are orthogonal to confidence. It will be of importance for future studies to disambiguate between a purely visual account and a motor account of these effects [29,34].

Recent work has shown that sharing confidence between individuals can improve group decision-making [1]. How this confidence sharing is carried out has remained unclear. One simple means of understanding another person's confidence is via explicit, metacognitive communication. But such communication may be noisy, and in some circumstances may actually reduce the performance benefit of sharing [35]. Another perspective is that confidence could be directly ‘read out’ from another individual's decisions without explicit communication [2]. Here, we provide a mechanism for which this implicit readout might occur. By relying on the relationship between movement kinematics and subjective confidence, individuals can harness action observation to infer another individual's level of subjective confidence. Future studies could usefully examine whether this mechanism contributes to the wisdom of crowds. More generally, our results support an intimate link between decision confidence and the temporal dynamics of the decision itself [36].

One of the central planks of the motor simulation theory is that action understanding is critical for social interaction [12]. Indeed, a dominant view is that deficits in social interaction are in part owing to a failure of correct motor simulation ([37–39], but see [40] for an alternative view). As mentioned previously, the majority of this research has focussed on the inference of goals and intentions. The results here show that subjective states are manifest in subtle changes in the way in which an action is executed. One possibility is that use of our own motor system to simulate the kinematics of an observed action permits estimation of someone else's higher-order cognitive states—confidence [3], values [41] and emotions [42,43]—simply by observing their actions. Having access to this information is of clear importance in social interactions, perhaps even more so than being able to infer the intended goal or intention of an observed action.
